# Habituation to thaxtomin A in hybrid poplar cell suspensions provides enhanced and durable resistance to inhibitors of cellulose synthesis

**DOI:** 10.1186/1471-2229-10-272

**Published:** 2010-12-10

**Authors:** Viviane Brochu, Marie Girard-Martel, Isabelle Duval, Sylvain Lerat, Gilles Grondin, Olivier Domingue, Carole Beaulieu, Nathalie Beaudoin

**Affiliations:** 1 Natural Resources Canada, Canadian Forest Service, Pacific Forestry Centre, Victoria, BC, Canada V8Z 1M5; 2 Natural Resources Canada, Canadian Forest Service, Laurentian Forestry Centre, Québec, QC, Canada G1V 4C7; 3 Centre SÈVE, Département de biologie, Faculté des Sciences, Université de Sherbrooke, Sherbrooke, QC, Canada J1K 2R1

## Abstract

**Background:**

Thaxtomin A (TA), a phytotoxin produced by the phytopathogen *Streptomyces scabies*, is essential for the development of potato common scab disease. TA inhibits cellulose synthesis but its actual mode of action is unknown. Addition of TA to hybrid poplar (*Populus trichocarpa *x *Populus deltoides*) cell suspensions can activate a cellular program leading to cell death. In contrast, it is possible to habituate hybrid poplar cell cultures to grow in the presence of TA levels that would normally induce cell death. The purpose of this study is to characterize TA-habituated cells and the mechanisms that may be involved in enhancing resistance to TA.

**Results:**

Habituation to TA was performed by adding increasing levels of TA to cell cultures at the time of subculture over a period of 12 months. TA-habituated cells were then cultured in the absence of TA for more than three years. These cells displayed a reduced size and growth compared to control cells and had fragmented vacuoles filled with electron-dense material. Habituation to TA was associated with changes in the cell wall composition, with a reduction in cellulose and an increase in pectin levels. Remarkably, high level of resistance to TA was maintained in TA-habituated cells even after being cultured in the absence of TA. Moreover, these cells exhibited enhanced resistance to two other inhibitors of cellulose biosynthesis, dichlobenil and isoxaben. Analysis of gene expression in TA-habituated cells using an Affymetrix GeneChip Poplar Genome Array revealed that durable resistance to TA is associated with a major and complex reprogramming of gene expression implicating processes such as cell wall synthesis and modification, lignin and flavonoid synthesis, as well as DNA and chromatin modifications.

**Conclusions:**

We have shown that habituation to TA induced durable resistance to the bacterial toxin in poplar cells. TA-habituation also enhanced resistance to two other structurally different inhibitors of cellulose synthesis that were found to target different proteins. Enhanced resistance was associated with major changes in the expression of numerous genes, including some genes that are involved in DNA and chromatin modifications, suggesting that epigenetic changes might be involved in this process.

## Background

Thaxtomin A (TA) is the main phytotoxin produced by the pathogen *Streptomyces scabies*, the most important causal agent of potato common scab [1,2]. Production of TA is required for the development of disease symptoms [1,3-5], and application of the purified toxin on immature potato tuber tissues induces the production of scab-like lesions [6]. A wide variety of plant species are sensitive to exogenous application of TA, inducing symptoms ranging from growth inhibition, root stunting, and cell hypertrophy to cell death [3,4,7]. TA can also activate a genetic program of cell death in *Arabidopsis thaliana *cell suspensions [8].

Previous reports have shown that TA inhibits crystalline cellulose biosynthesis [9]. Recent evidence indicates that addition of TA to Arabidopsis seedlings decreased the stability of cellulose synthase (CESA)-complexes, releasing them from the plasma membrane to be accumulated in small microtubule-associated compartments [10]. This is similar to what has been described in response to another inhibitor of cellulose synthesis, isoxaben (IXB) [11]. Moreover, changes in gene expression induced in response to TA or IXB treatment were very similar, indicating that the mode of action of TA closely resembles that of IXB [10,12]. While mutant analyses suggest that IXB targets CESA3 and CESA6 [13,14], the mode of action and specific target of TA have not yet been identified.

The plant cell wall is important to maintain cell shape and strength in response to the high turgor pressure applied by the vacuole. Cellulose, the main glycan component of the plant cell wall, is organized into microfibrils, which are bound by hemicelluloses to form a network embedded in a matrix of pectins [15]. This strong but flexible arrangement of complex polysaccharides is important not only for the control of plant cell structure, expansion and position, but is also involved in several cellular processes, including cell differentiation, intercellular communication and defense responses [15,16]. The composition and organization of the plant cell wall change during the plant cell cycle, growth, differentiation and can be altered in response to biotic and abiotic stress [e.g., [17-23]]. Previous reports have demonstrated the possibility of adapting or "habituating" plant cells to grow and divide in the presence of inhibitors of cellulose synthesis, such as IXB and dichlobenil (DCB) by adding incremental concentrations of the inhibitors over several cell generations [24-32]. While some variations were noted between different plant species, habituation was generally associated with a decrease in cellulose that was compensated by changes in the composition or organization of the cell wall, where the xyloglucan-cellulose network was partly or almost completely replaced by pectins. Likewise, plant cell cultures habituated to water and salt stresses presented modified cell walls with a decrease in cellulose content with increases in hemicellulose and proteins and a general reorganization of the pectin network [18,19]. Gene expression analyses in hormone habituated cells, which are capable of unlimited growth in the absence of cytokinins, also suggested that this type of habituation was associated with changes in cell wall biochemistry [33]. Reciprocally, mutations perturbing cellulose synthesis or cell adhesion, as in the mutants *tsd1/KORRIGAN *[34,35] and *tsd2 *[36,37] respectively, led to hormonal habituation. These data demonstrate that there is a reciprocal link between the physiological, developmental or metabolic state of the cell and the composition of its cell wall.

In this work, we show that while inhibition of cellulose synthesis by TA can activate cell death in hybrid poplar cells, it is also possible to habituate poplar cell suspensions to grow and divide in the presence of lethal levels of TA. Habituation to TA was associated with modifications in the cell wall composition, with a decrease in crystalline cellulose and an increase in pectins. Interestingly, we found that TA-habituated cells cultured in the absence of TA have remained resistant to TA for more than three years. Remarkably, these cells also exhibited enhanced resistance to two other inhibitors of cellulose synthesis, IXB and DCB, and this resistance has been sustained for more than three years. To investigate the genetic mechanisms that are involved in establishing and maintaining resistance to TA, we have performed a global transcriptional analysis in TA-habituated cells cultured in the absence of TA.

## Results and Discussion

### Effects of TA on hybrid poplar cell suspensions

**Figure 1:**
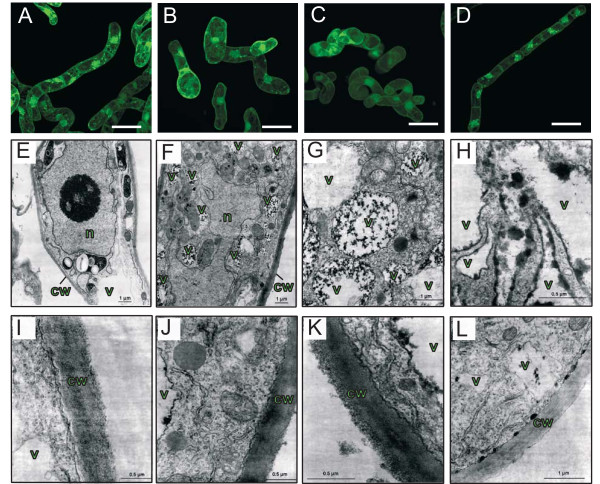
**Morphological changes in hybrid poplar suspension-cultured cells treated with TA and habituated to TA**. **A-D **Confocal microscopy imaging of hybrid poplar cells stained with fluorescein diacetate: **A **treated with methanol for 24 h; **B **treated with TA (1.0 μM) for 24 h; **C **habituated to 1.7 μM TA; **D **TA(-)hab cells. Bar = 50 μm. **E-L **Electron microscopy imaging of 5-day-old non-habituated hybrid poplar cells (**E **and **I**) and 5-day-old TA(-)hab cells (**F-H**, **J-L**). n = nucleus; cw = cell wall; v = vacuole.

**Figure 2:**
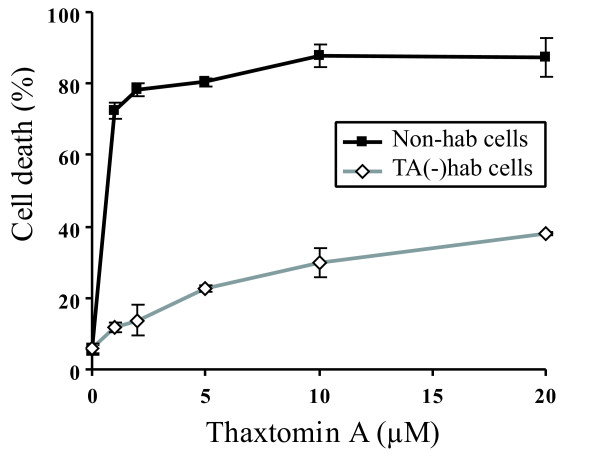
**Induction of cell death by TA in hybrid poplar suspension-cultured cells and TA(-)hab cells**. Percentage of dead cells detected by trypan blue staining in hybrid poplar suspension cultures (Non-hab cells) and TA(-)hab cells treated with the indicated concentrations of TA for 48 h. The values represent the means ± SD of three independent experiments including at least 500 cells each.

It was shown previously that TA induced an increase in cell volume in tobacco suspension cultures [7] and in Arabidopsis cells [8]. Similarly, some of the hybrid poplar suspension-cultured cells treated with 1.0 μM TA for 24 h were hypertrophied when compared to control cells treated with methanol (Figure 1A-B). However, the increase in cell volume was less pronounced in poplar cells than in Arabidopsis cells. Similar changes were also observed when adding IXB (5.0 μM) or DCB (5.0 μM) (data not shown). As reported for Arabidopsis cell suspensions [8], TA induced cell death in poplar suspension cultures; 73% of the cells were dead 48 h after adding 1.0 μM TA (Figure 2). Cell death in poplar cells was also associated with nuclear DNA fragmentation, a typical hallmark of programmed cell death (PCD), as detected by the TUNEL assay (Additional file 1 Fig. S1).

TA has been shown to inhibit the incorporation of radioactive glucose in the acid-insoluble fraction of the cell wall, which corresponds to crystalline cellulose [9]. The effects of TA on the level of crystalline cellulose in poplar cells were analyzed by quantifying glucose in the acid-insoluble fraction of the cell walls. As indicated in Table 1, cells in contact with TA for 24 h contained 12% less crystalline cellulose than control cells. These results indicated that TA rapidly inhibited the synthesis or incorporation of cellulose in the poplar cell walls, demonstrating that TA can also alter cellulose synthesis in a tree species.

**Table 1:** Quantification of glucose in cell walls1

Treatment	Glucose (μg mg^-1 ^dry wall)		
	
	Total glucose	Acid-insoluble fraction	Soluble fraction^c^
MeOH	344.5 ± 12.9^a^	296.1 ± 8.1^a^	48.4
TA	328.4 ± 22.0^b^	259.7 ± 2.5^b^	68.7

^1^Results were obtained in total cell walls and in the acid-insoluble (crystalline cellulose) fraction 24 h after adding TA in cells grown for 5 days after subculture. Results are the means ± SD of three independent experiments. Treatment: MeOH = hybrid poplar cells + methanol; TA = hybrid poplar cells + TA (1.0 μM).

^a, b ^Statistically different values (Student's *t*-test, *P*< 0.05) are indicated with a different letter in a column for each experiment.

^c ^Values were obtained by subtracting the acid-insoluble fraction values from the whole cell walls values.

### Habituation of poplar cell suspensions to TA is associated with changes in cell wall composition

Plant cell habituation to inhibitors of cellulose synthesis such as DCB and IXB has been reported [24,26,28,30,32]. To habituate hybrid poplar cell suspensions to TA, we initially cultured them with a low level of TA (0.1 μM) that was gradually increased up to 1.3 μM over a period of 12 months. These cells became resistant to lethal TA concentrations. During the process of habituation, changes in cell morphology and growth rate were observed. When compared to non-habituated cells, TA-habituated cells were wider, rounder, twisted and formed aggregates (Figure 1C). Their growth rate was also greatly reduced. In order to have a volume of cell inoculum similar to that of control cells, subculture of TA-habituated cells had to be performed every other week instead of weekly. TA-habituated cells were then subcultured in the absence of TA for at least 18 months before performing additional characterization. This procedure had been termed "dehabituation" in previous work [31] but TA-dehabituated cells will be further referred to as "TA(-)hab" cells. As observed in other habituated cells, TA(-)hab cells had a modified cell volume and reduced growth rate but they progressively became more elongated and did not form aggregates (Figure 1D; Additional file 1 Table S1). Electron microscope analysis also revealed the accumulation of electron-dense material in fragmented vacuoles (Figure 1F-H) and in some cases close to cell walls (Figure 1L). Cell walls of TA(-)hab cells appeared as thick as those of control cells but were more opaque (Figure 1J-K).

Habituation was associated with changes in the cell wall composition. The proportion of the various monosaccharides evaluated in this work, including glucose, in relation to the total sugars (Table 2), was not significantly different in both types of cells (Additional file 1 Fig. S2). However, TA(-)hab cell walls contained about 25% less glucose in the crystalline cellulose fraction (acid-insoluble fraction) than non-habituated cell walls. In addition, the overall level of glucose in the cell wall material was significantly reduced in TA(-)hab cells, while the estimated level of glucose remaining in the acid-soluble fraction was increased. This fraction is mainly composed of xyloglucans, non-crystalline β-1,4-glucans and pectins [9], thus supporting a general reorganization of the cell wall to compensate for the reduction in cellulose. The level of uronic acids was determined in the CDTA-soluble pectin fraction of dry cell walls. The value increased from 17.1 μg to 31.4 μg mg^-1 ^cell wall in TA(-)hab cells, representing 1.8 times more CDTA-soluble pectins than in the non-habituated cell walls. Microscopic analysis using ruthenium red for staining of pectic polysaccharides also revealed a more intense staining in the cell walls of TA(-)hab cells compared to a very faint staining in control cells, also suggesting the accumulation of more pectins in the cell walls of TA(-)hab cells (Additional file 1 Fig. S3).

**Table 2:** Quantification of sugars in cell walls1

Cell type	Total sugars (μg mg^-1 ^dry wall)	Glucose (μg mg^-1 ^dry wall)			Uronic acids^d^ (μg mg^-1 ^dry wall)
			
		Total glucose	Acid-insoluble fraction	Soluble fraction^c^	
Non-hab	755.0 ± 113.0	445.6 ± 27.5^a^	384.1 ± 15.0^a^	61.5	17.1 ± 3.0^a^
TA(-)hab	640.1 ± 50.0	391.9 ± 3.6^b^	287.2 ± 53.1^b^	104.7	31.4 ± 3.2^b^

^1^Total sugars, glucose and uronic acids (CDTA fraction) were quantified in dry cell walls from different cell types. Glucose was also quantified in the acid-insoluble fraction (crystalline cellulose). Samples were taken 10 days after subculture. Results are the means ± SD of three independent experiments. Cell type: Non-hab = non-habituated hybrid poplar cells; TA(-)hab = TA-habituated cells without TA.

^a, b ^Statistically different values (Student's *t*-test, *P*< 0.05) are indicated with a different letter in a column for each experiment.

^c ^Values were obtained by subtracting the acid-insoluble fraction values from the whole cell walls values.

^d ^Uronic acids were quantified from the CDTA-soluble pectin fraction.

Habituation to inhibitors of cellulose synthesis has frequently been associated with changes in the composition and organization of the cell wall characterized by a decrease in cellulose content and an increase in the pectin network [24,26,28,30,38,39]. However, the extent to which the cell wall was modified varied widely between habituated cells depending on the species and inhibitor used. In TA(-)hab cells, the decrease in crystalline cellulose was much less substantial than that reported in bean cells habituated to IXB [28] or tomato cells habituated to DCB [24], where close to 72% and 97% reduction was observed respectively. This may be due to the fact that each inhibitor uses a different mode of action to inhibit cellulose synthesis. It was also proposed that variations in the initial composition of the cell wall in different species could influence cell wall adaptations during the habituation process [25,26].

### TA(-)hab cells are more resistant to TA, DCB and IXB

**Figure 3:**
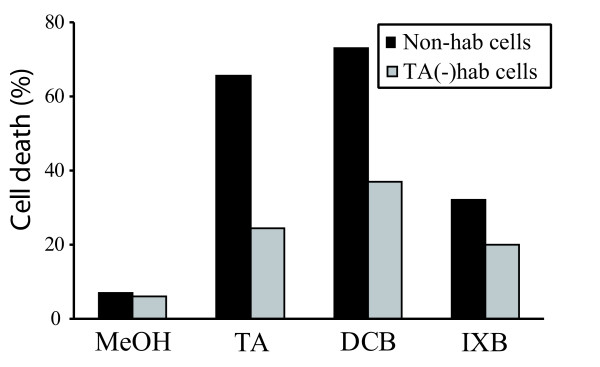
**Induction of cell death by inhibitors of cellulose synthesis**. Percentage of dead cells detected by trypan blue staining in hybrid poplar suspension cultures (Non-hab cells) and TA(-)hab cells treated with methanol (MeOH) as a control or with 2.0 μM TA, 5.0 μM DCB or 5.0 μM IXB for 48 h. At least 500 cells were counted for each treatment. The values are representative of four independent experiments.

Resistance to TA was tested in TA(-)hab cells. Even after being subcultured in the absence of TA for more than three years, TA(-)hab cells still tolerated high levels of TA (Figure 2). Cell death was below 14% in the presence of 2.0 μM TA for 48 h compared to 78% for non-habituated cells. In the presence of 20 μM TA, the level of cell death reached 38% for TA(-)hab cells while 87% of non-habituated cells were dead. TUNEL assays performed on TA(-)hab cells treated with TA also indicated that DNA fragmentation was increasing in dying cells, suggesting that PCD was still activated in response to TA (Additional file 1 Fig. S1 G-I). These results suggest that a sub-population of TA(-)hab cells remained susceptible to TA. Because the modified composition of the cell walls of TA(-)hab cells was reminiscent of that of DCB- and IXB-habituated cells, TA(-)hab cells were tested for resistance to these inhibitors. A concentration of 5.0 μM was used for IXB as poplar cells were more tolerant to this inhibitor than other species, with a level of cell death lower than 40% after a 48 h-treatment with 5.0 μM IXB compared to about 45% of cell death after a 48 h-treatment with 100 nM IXB in *Arabidopsis thaliana *[12]. Induction of cell death after treatment with DCB or IXB was always less pronounced in TA(-)hab cells when compared to non-habituated cells in all four assays over a three-year period. As shown in Figure 3, more than 72% of hybrid poplar cells were killed by DCB after 48 h compared to 37% in TA(-)hab cells. IXB treatment induced 32% of cell death in hybrid poplar cells compared to 19% in TA(-)hab cells. Hence, habituation to TA not only provided specific resistance to the TA toxin itself but also enhanced cell survival in response to two other molecules also known to inhibit cellulose synthesis. Therefore, it is unlikely that resistance to TA is simply due to a detoxification mechanism that would transform TA to less toxic metabolites, as it was reported in the presence of the fungus *Aspergillus niger *[40]. Such a specific mechanism could not operate on structurally different molecules such as DCB and IXB. It is also unlikely that enhanced resistance in TA(-)hab cells would be due to a modification of the inhibitors' target, since each inhibitor is thought to perturb cellulose synthesis by targeting specific molecules, with IXB possibly targeting CESA subunits 3 and 6 [13,14], and DCB proposed to target either a small protein of 12-18 kD [41] or the microtubule-associated protein MAP20 [42]. In any cases, habituation to TA most probably activated a mechanism that enhanced resistance to inhibition of cellulose synthesis per se rather than enhancing resistance to the inhibitory molecules themselves.

Since TA-, DCB- and IXB-habituated cells all presented a modified cell wall composition where pectins accumulated to compensate for reduced cellulose level, it is tempting to speculate that enhanced resistance to inhibition of cellulose synthesis was due to cell wall adaptations that occurred during habituation. As found for TA(-)hab cells, it was reported that DCB-habituated bean cells cultured in the absence of DCB for several months (DCB-dehabituated cells) were still resistant to lethal levels of DCB [38,43]. The fact that dehabituated cells retained a high level of resistance even when cultured in the absence of the inhibitor supports previous reports suggesting that a durable mechanism is activated during the habituation process [26,30,38]. However, while DCB-dehabituated cells were still resistant to DCB, the composition of their cell walls was progressively restored close to control levels after being cultured in absence of DCB for more than 6 months, retaining a higher proportion of pectins with lower degree of methyl-esterification than in habituated cells [31,38,44]. This contrasts with TA(-)hab cells which had a reduced cellulose content even when cultured for more than 18 months in the absence of TA. This suggests that the major changes in cell wall composition, such as reduced cellulose and increased pectins, were not required for resistance to DCB. Garcia-Angulo et al. (2009)[43] have proposed that the cellulose synthesis machinery in DCB-dehabituated cells would be less effective but more resistant to DCB. Mutations affecting the cellulose biosynthesis machinery could be responsible for the enhanced and durable resistance to DCB in those cells [43]. It is possible that mutations in components of the cellulose synthesis machinery could lead to defective cellulose synthesis in TA(-)hab cells. However, it is less likely that these mutations would lead to an increased tolerance to different inhibitors of cellulose synthesis. Further investigations will be required to determine whether reduced cellulose synthesis in TA(-)hab cells is caused by mutations affecting the cellulose synthesis machinery or due to the activation of a mechanism of adaptation to inhibition of cellulose synthesis.

### Habituation to TA is associated with important transcriptional changes

**Figure 4:**
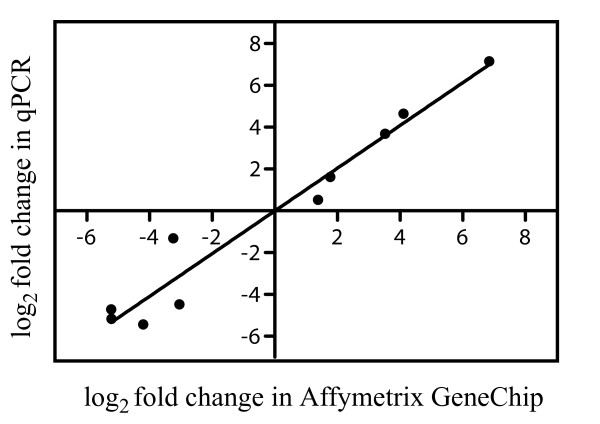
**Validation of microarray results by qPCR**. Log_2 _average fold-change from Affymetrix GeneChip data plotted with log_2_-transformed qPCR fold-change in TA(-)hab cells for five upregulated and five downregulated genes. qPCR data represent the mean value obtained from three independent replicates that were repeated twice.

To study the genetic mechanisms that may be involved in TA resistance and in maintaining this resistance in TA(-)hab cells, we have performed a global transcriptional analysis in TA(-)hab. While transcriptional changes do not directly represent the overall physiological or metabolic state of plant cells, modifications in gene expression provide good indications on how plant cells respond to changing environments and how these responses are sustained at the gene expression level. Microarray analysis was carried out using the Affymetrix GeneChip Poplar Genome Array. Data were normalized and analyzed by Robust Multi-Array Average (RMA) [45] using the FlexArray software [46]. Probesets with a more than 2.5-fold change (FC) in expression in TA(-)hab cells when compared to non-habituated cells and a *P *value ≤ 0.05 following significance analysis of microarrays (SAM) were selected as being up- or downregulated (Additional file 2 Table S2 and Additional file 3 Table S3). Overall, 404 probesets corresponding to 346 predicted genes were upregulated in TA(-)hab cells and 880 probesets associated with 764 predicted genes were downregulated. Validation of microarray results was performed using qPCR for five genes upregulated and five genes downregulated in TA(-)hab cells. As shown in Figure 4 and Additional file 4 Table S4, qPCR results were strongly correlated with the microarray data. Regression analysis of log_2_-transformed FC generated slope y = 1.022 - 0.0027 and R^2 ^= 0.9542 (*P *< 0.0001), demonstrating the high precision of the GeneChip Poplar Genome Array data.

**Figure 5:**
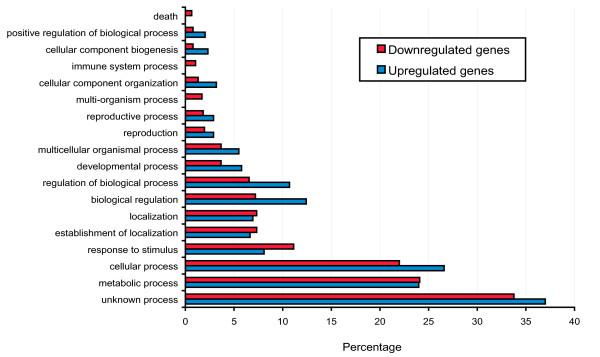
**Functional characterization of genes differentially expressed in TA(-)hab cells**. Proportion of biological process annotations using AgriGO for genes significantly downregulated >2.5 FC (red bars) or significantly upregulated >2.5 FC (blue bars) in TA(-)hab cells.

Candidate gene annotations were performed using PLEXdb [47], PopArray database [48] and the NetAffx from the Affymetrix website http://www.affymetrix.com as described in Methods. Gene products and functions were mainly predicted based on sequence homology. The names of predicted poplar genes were indicated when available. Otherwise, the putative function of the closest Arabidopsis homologous gene was indicated to facilitate comparison (Additional file 2 Table S2). Because the actual function of most poplar genes remains to be shown, some of the predicted functions may be incorrect as similar sequences may have different functions in diverse species. Gene ontology analysis was performed using the AgriGO analysis toolkit and database (Figure 5) [49]. Predicted genes that had no GO annotations (258 downregulated genes, 128 upregulated genes) were classified in the "unknown biological process" category. In downregulated genes (Figure 5), the most frequent annotations were related to metabolic process (24.1%, including 3.3% in secondary metabolic process), cellular process (22.0%), response to stimulus (11.1%, including 6.4% in the stress category), localization and transport (7.3%) and biological regulation (7.2%). These same categories were also highly represented in upregulated genes, with 24.0% annotations in metabolic process, 26.6% for cellular process (including 5.8% for transcription), 8.1% for response to stimulus (including 5.5% for response to stress) and 6.7% for localization and transport. Moreover, upregulated genes included 5 GO annotations (1.4%) for chromatin assembly or disassembly, in a reference group that contains only 79 genes.

### Comparison with other habituation experiments

**Figure 6:**
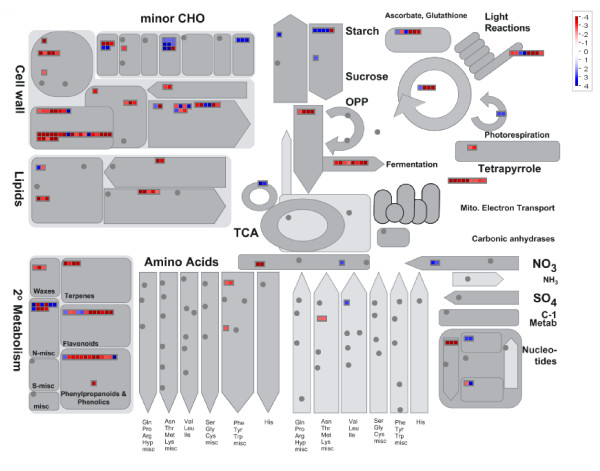
**Changes in expression for genes involved in metabolism**. MapMan overview of significant changes in expression (> 2.5 FC) for genes associated with metabolism in TA(-)hab cells.

In 2004, Manfield et al. have characterized global gene expression using the Affymetrix ATH1 GeneChip in Arabidopsis cells that were habituated to IXB [32]. These cells contained less glucose and more pectins in their cell walls. IXB-habituated (referred hereafter to "IXBhab") cells were still grown in the presence of IXB in contrast to TA(-)hab cells that were subcultured in the absence of TA. As mentioned earlier, there is experimental evidence suggesting that the mode of action of TA resembles that of IXB, although each molecule individually activates a few distinctive responses [10,12]. Hence, the identification of conserved patterns of gene expression in both experiments could help identify the mechanisms that are involved in providing resistance to inhibitors of cellulose synthesis. However, it is essential to keep in mind the important differences in species, growth conditions, method of habituation and type of microarray analyses when examining these results. In order to compare gene expression data in IXBhab cells with those of TA(-)hab cells, raw microarray data (CEL file) from IXBhab cells available at GEO (GSE6181) or NASC (NASCARRAYS-27) were analyzed using RMA and SAM with the Flexarray software. Genes that displayed a change of expression that was more than 2 FC and a *P *value ≤ 0.05 following SAM were selected for comparison (Additional file 5 Table S5). With this method, more genes were considered to be significantly up- or downregulated in IXBhab cells than previously reported, but the expression of genes already reported to be upregulated or downregulated followed the same trend [32]. Gene expression in TA(-)hab cells was first compared with data from IXBhab cells using the closest AGI predicted for each poplar probeset (Additional file 2 Table S2). However, since matching AGIs are predicted on the basis of sequence homology, it is possible that similar sequences may encode proteins with different functions and conversely, that divergent sequences encode proteins with similar functions. To overcome some of the difficulties in comparing gene expression between different species, we have chosen to use the MapMan software [50,51] to evaluate globally how different cellular processes and metabolic pathways are affected in TA(-)hab cells when compared to IXBhab cells. We assembled a MapMan mapping file based on expression data from TA(-)hab cells using the poplar Ptrich_AFFY_09 mapping file that was updated with information from the most recent annotation. MapMan results for "Metabolism overview" are presented in Figure 6 for TA(-)hab cells and in Additional file 1 Fig. S4 for IXBhab cells. Results for "Regulation overview" and "Cellular response" are presented in Additional file 1 Fig. S5 and S6. Differential gene expression was observed in cell wall synthesis and modification pathways as well as in secondary metabolism, with more genes downregulated in TA(-)hab cells than in IXBhab cells. A notable difference was in the photosynthesis process, where several genes were upregulated in IXBhab cells with little changes in gene expression in TA(-)hab cells. We speculate that different growth conditions may explain this difference, as TA(-)hab cells were grown in the dark, and we suspect that IXBhab cells were grown in light, although this has not been stated. To facilitate comparison, we have also used MapMan to generate a list of differentially expressed genes in IXBhab cells that are classified according to the major BinCode functional categories (Additional file 5 Table S5).

### Expression of cell wall-related genes

TA(-)hab cells have a modified cell wall, with less cellulose and more pectins. To help determine how TA(-)hab cells adjusted their cell wall composition, we have looked more closely at the expression of genes involved in cell wall synthesis, modification or degradation corresponding to the BinCode category 10 (Additional file 2 Table S2). Most predicted genes belonging to this category were downregulated. Cellulose is synthesized by large membrane complexes constituted by CESAs [52]. Expression of *CESA *genes was not significantly modified by more than 2.5 FC in TA(-)hab cells. Only one predicted *CESA*-like gene (predicted ortholog of *CSLG3*) was downregulated. Hence, the reduced cellulose content was not associated with differential expression of cellulose synthase genes, as it was reported for IXBhab cells [32]. However, since there is increasing evidence that CESA complexes are associated with other proteins that aid microfibril formation and that link the complexes to nearby microtubules for guidance along the membrane [15], it is possible that expression of genes encoding some of these unidentified proteins could be altered in TA(-)hab cells. Other downregulated genes included genes encoding proteins involved in cell wall degradation (glycosyl hydrolase, xyloglucan endotransglucosylases/hydrolases (XTH), polygalacturonases), cell wall modification (polygalacturonases, pectin(acetyl)esterases, XTHs) and cell wall proteins (fasciclin-like arabinogalactan-proteins and extensins). Only a few genes were upregulated, such as genes predicted to encode beta-xylosidases, a beta-mannan endohydrolase, a polygalacturonase, a pectinesterase, two expansins and a lyase.

Expression data in TA(-)hab cells was compared to that of Arabidopsis IXBhab cells [32] using matching AGIs (Additional file 2 Table S2 and Additional file 5 Table S5). Several genes encoding predicted orthologs had a similar pattern of expression in both cell types, except for two XTHs (*XTH9 *and *XTR7*), one pectinacetylesterase and one polygalacturonase inhibiting protein gene (*PGIP1*) that were upregulated in IXBhab cells. Moreover, a callose-synthase gene (*CALS1*) downregulated in IXBhab cells was upregulated in TA(-)hab cells. However, two other callose synthase genes (*AtGSL09 *and *AtGSL12*) were upregulated in IXBhab cells. Several predicted cell wall-related poplar genes differentially expressed in TA(-)hab cells did not have a matching Arabidopsis gene differentially regulated in IXBhab cells. However, these poplar genes had a predicted function that was similar to that of at least one of the genes that were differentially expressed in IXBhab cells. For instance, a proline-rich extensin like gene downregulated in TA(-)hab cells was also downregulated in IXBhab cells. Therefore, TA(-)hab and IXBhab cells exhibited similar changes in the expression of a large overlapping set of genes involved in cell wall modifications, even though TA(-)hab cells were no longer cultured in the presence of TA. Moreover, this analysis shows that despite species differences, it is possible to correlate expression data in TA(-)hab poplar cells with those of IXBhab Arabidopsis cells, at least at the level of cell wall-related genes. It would certainly be of interest to determine whether similar transcriptional changes also occurred in DCB-habituated cells. This could eventually help pinpoint a potential conserved mechanism of adaptation to inhibition of cellulose synthesis. On the other hand, we suspect that most of these changes would be lost during the DCB-dehabituation process since the cell wall composition was then restored close to initial levels [31,38]. Nonetheless, some modifications were retained in DCB-dehabituated cells, such as a reduced level of arabinogalactan proteins and the accumulation of modified pectins [31,38]. We found that some genes predicted to encode arabinogalactan proteins and pectin modifying enzymes were downregulated by more than 2.5 FC in TA(-)hab cells, suggesting that less arabinogalactan proteins and pectin modifications were present in the TA(-)hab cell walls. The implication of these modifications for the establishment of durable resistance to inhibitors of cellulose remains to be shown.

### Genes involved in the phenylpropanoid pathway

The phenylpropanoid pathway leads to the synthesis of a wide range of natural products in plants, including lignans, lignin, flavonoids and anthocyanins, several of which are induced by stress [53]. In poplar, genes involved in the synthesis of phenylpropanoids are part of expanded families that contain genes with conserved functions as well as new members whose biochemical function may be distinct [54-56]. Several genes predicted to belong to these large gene families were downregulated in TA(-)hab cells. These include genes predicted to encode one cinnamyl-alcohol dehydrogenase (*CAD14*), one caffeic acid/5-hydroxyferulic acid O-methyltransferase (*COMT6*), two trans-caffeoyl-CoA 3-O-methyltransferases (*CCoAOMT1 *and *2*), and three different hydroxycinnamoyl-Coenzyme A shikimate/quinate hydroxycinnamoyltransferases (*HCT2*, *HCT5 *and *HCT7*). The poplar CCoAOMT1 and 2 have been shown to be specifically involved in lignin synthesis, as reduced CCoAOMT activity in poplar led to reduced lignin synthesis [56]. Lignin is deposited in the secondary cell walls to provide rigidity and impermeability to the cells. It is possible that reduced expression of these genes in TA(-)hab cells also turns down the production of lignin. However, *HCT2*, *5 *and *7*, as well as *COMT6 *and *CAD14*, are barely expressed in lignifying tissues, suggesting that they may be involved in other processes [55,56]. While ectopic lignification was observed in mutants with reduced cellulose synthesis [57] and in Arabidopsis seedlings treated with TA or IXB [10], IXBhab cells did not show any ectopic lignificaton [32]. Supporting these results, several genes specifically involved in lignin synthesis (BinCode 16, Additional file 5 Table S5) were also downregulated in Arabidopsis IXBhab cells, such as genes encoding a CCoAMT, a caffeic acid/5-hydroxyferulic acid *O*-methyltransferase (*AtOMT1*), a cinnamoyl CoA reductase (*CCR2*) and a cinnamyl-alcohol dehydrogenase 4 (*CAD4*).

Flavonoids function as sunscreen and as defense compounds and have been shown to accumulate in response to various stresses [58,59]. Some genes involved in the synthesis of flavonoids were also downregulated in TA(-)hab cells. These genes were predicted to encode a chalcone synthase (*CHS6*), which is the committed step to flavonoid synthesis, a flavonol synthase (*FLS*), which participates in the synthesis of flavonols, and an anthocyanidin reductase (*ANR/BAN1*), which is involved for the formation of proanthocyanidins [55,59]. However, the specific function of each isoform remains to be shown.

In poplar, several genes of the lignin and flavonoid synthesis pathways were dramatically upregulated during infection by *Melampsora medusae *leaf rust [60,61]. In contrast, gray poplar roots exposed to hypoxic stress displayed a reduced expression in lignin and flavonoid synthesis-related genes [62]. It was proposed that repression of the phenylpropanoid pathway in these conditions would be a way of inhibiting energy demanding mechanisms in favor of glycolysis to maintain carbon and energy metabolism in periods of O_2 _deficiency [62]. Similarly, downregulation of lignin and flavonoid synthesis pathways in TA(-)hab cells may help repress high energy consuming pathways to redirect carbohydrates to other processes that may be required for cell survival in response to reduced cellulose synthesis. However, while the metabolic outcome of repressing these pathways is unknown, we suspect that a significant fraction of the phenylpropanoids produced will not be incorporated in lignin and flavonoids and could either be accumulated or directed to other pathways. Accumulation of phenolics in vacuoles has been frequently reported [63]. It is possible that the electron dense material that was observed in vacuoles of TA(-)hab cells (Figure 1) were phenylpropanoids that accumulated due to repressed lignin and flavonoid synthesis, but this hypothesis remains to be tested. Whether these changes were related to enhanced resistance to TA is unknown at this time. While some of the genes involved in lignin synthesis were also dowregulated in IXBhab cells, we observed very limited changes in the expression of flavonoid synthesis-related genes, suggesting that modulation of this pathway may either be a specific response to TA or related to species differences in response to inhibition of cellulose synthesis.

### Expression of cell death-related genes

We have shown previously that TA and IXB activate a program of cell death in Arabidopsis cell suspensions [8] and in poplar (this work). Since TA(-)hab cells were able to survive in high concentrations of TA, it is possible that genes encoding proteins involved in regulating the onset of cell death were differentially regulated in TA(-)hab cells. We had found in previous work that more than half of the genes that were upregulated in common after a short exposure of Arabidopsis cells to TA or IXB were downregulated in IXBhab cells, suggesting that some stress-related mechanisms were turned down in those cells [12]. Interestingly, several genes predicted to control the process of cell death were differentially regulated in TA(-)hab cells. For example, a gene predicted to be the ortholog of *STP13*, which encodes a hexose transporter whose expression is correlated with PCD [64] was downregulated in TA(-)hab cells (FC -3.9). Another gene predicted to encode an ortholog of the Arabidopsis *DMR6 *was drastically downregulated in TA(-)hab cells (FC -37.5) and in IXBhab cells (FC -14.6). This gene has been shown to play a role in the onset of PCD during plant-pathogen interactions. Hence, absence of *DMR6 *in the Arabidopsis mutant *dmr6 *led to resistance to *Hyaloperonospora parasitica *that was associated with the absence of PCD and reactive oxidative intermediates with no induction of the expression of the defense-associated gene *PR-1 *[65]. Several other defense-related genes were downregulated in TA(-)hab cells, including numerous disease resistance proteins that may play a role in the regulation of the hypersensitive cell death [66].

Another set of genes predicted to function in protecting against cell death was upregulated in TA(-)hab cells. These include a gene putatively encoding a spermine synthase orthologous to the Arabidopsis *ACAULIS5 *(*ACL5*) gene that was upregulated 6.5 times in TA(-)hab cells (7.1 in IXBhab cells). Mutant analysis has shown that ACL5 is involved in xylem specification. Expression of ACL5, a spermine synthase, is thought to prevent premature death of the developing vessel element [67]. This is corroborated by the fact that exogenous application of spermine can prolong xylem element differentiation while stimulating cell expansion and cell wall elaboration. Another gene was the predicted poplar gene encoding an ortholog of *AtBAG6 *(upregulated 2.8 times), a member of BAG family proteins also believed to be involved in cell survival [68]. It is possible that differential regulation of cell genes regulating the PCD that is induced in response to TA could significantly contribute to cell survival in TA(-)hab cells.

### Expression of genes involved in cell cycle

Several genes predicted to be involved in the control of cell division and cell cycle (Bincode 31.2 and 31.3) were upregulated in poplar TA(-)hab cells as well as in Arabidopsis IXBhab cells (Additional file 1 Fig. S5 and S6; Additional file 2 Table S2 and Additional file 5 Table S5). These include genes predicted to encode for the cyclin-dependent kinase CDKB1;2, which accumulates in a cell cycle-dependant manner to reach a maximum level at the G2/M transition where its activity is required [69]; the cyclin-dependent kinase regulators, CYCB2;4, CYCB1:4, whose expression also peaks at the G2/M transition and during M phase transition; and the cell division cycle-like protein CDC45 that accumulates in the G1/S transition [70]. Other members were also upregulated in IXBhab cells, e.g. *CYCB2;2*, *CYCD3;1*, *CYCB1;4 *and *CYCB2;1*. Cellulose synthesis fluctuates during the cell cycle, as it is required for cell elongation, differentiation and cell plate formation. It was shown that cellulose is deposited in cell plates at the late M phase after callose deposition [71]. Results obtained in the dinoflagellate *Crypthecodinium cohni *have suggested that cell cycle progression is coupled with cellulose synthesis at the G1 phase [72]. Hence, inhibition of cellulose synthesis would halt cell growth by introducing a G1 cell cycle delay that could lead to a cell cycle arrest in late M phase [72]. Upregulation of cell cycle-related genes in TA(-)hab and IXBhab cells may be a consequence of the reduced cellulose content, which in turn could signal changes in the progression of the cell cycle.

### Expression of genes involved in DNA and chromatin modifications

Another important feature of TA(-)hab cells was their capacity to remain resistant to TA over several generations. Therefore, most of the changes in gene expression that were induced during the habituation process and that are important for resistance to TA must be conserved after cell division. Mitotically transmitted changes in gene expression can be caused by direct and irreversible alterations in the original DNA sequence (mutations) or may be mediated by epigenetic processes, such as reversible DNA methylation, histones modifications and chromatin remodeling [73]. It is known that both mutations and epigenetic modifications are more frequently induced during plant tissue culture than in whole plants [74]. Work by Pishke et al. (2006) [33] has shown that hormone habituation of Arabidopsis cells was associated with transcriptional activation of epigenetic-related genes involved in DNA methylation, histone methylation and deacetylation, as well as chromatin remodeling factors. DNA and chromatin modifications occurring during hormone habituation may be critical for the acquisition of cytokinin habituation. In TA(-)hab cells, several poplar genes predicted to encode histones were upregulated, including histone H1 (*HON901*), H2 (*HTA902/HTA912*), H3 (*HTR910/HTR914*) and H4 (*HFO905/HFO907*), except for the gene predicted to encode the histone variant H1.2 that was downregulated. Histone proteins are important for nucleosome and chromatin formation. In particular, histone variants may be important for specialized functions as their incorporation at certain regions of the chromosomes may confer specific structural or functional features to chromatin [75]. Arabidopsis orthologs of several of these genes were also found to be significantly upregulated (> 2.0 FC) in hormonally habituated cells (*H2A*, *H2B*, *H3*, *H3.2 *and *H4*) and in IXBhab cells (*H2A*, *H2A.Z*, *H2B*, *H3*), but downregulation was also observed for histone H1. Several genes participating in DNA and chromatin modifications were also found to be upregulated in TA(-)hab cells, including genes predicted to encode: a cytosol-specific methyltransferase (*DMT909*) which is involved in DNA methylation (FC 2.7); high-mobility-group HMGA (FC 3.2) and HMGB (FC 2.6) proteins, which are members of chromatin-associated proteins that would act as architectural factors in nucleoprotein structures and which regulate DNA-dependent processes including transcription [76]; a chromatin remodeling complex subunit (*CHR942*) that is a member of SNF2 domain-containing protein family (FC 2.8), which includes proteins that are proposed to play a role in gene silencing and that would interact with histone variants to alter chromatin structure [75]; a trithorax-related protein/SET-domain containing protein (*SDG933*; FC 2.5) whose Arabidopsis predicted ortholog TXR5 was shown to encode a H3K27 monomethyltransferase that is required for gene silencing through histone methylation [77].

Changes in DNA methylation patterns and chromatin modification events have also been correlated with activation of transposons [rev. in [78,79]], as it was observed in hormone habituated cells [33]. However, we did not detect differential expression of transposon-related sequences in TA(-)hab cells. It may be that differential expression of transposon-related sequences took place in TA(-)hab cells but at a level that was below 2.5 FC. Alternatively, activation of transposons could have occurred at an earlier stage of the habituation process (e.g., in the presence of TA) to be silenced later on due to epigenetic modifications [79].

However, activation of transposons was clearly induced in IXBhab cells that were still cultured in the presence of IXB (Additional file 5 Table S5). At least 10 transposon-related sequences, including copia-like retrotransposons, gypsy-like retrotransposons and a CACTA-like transposase family were differentially regulated in IXBhab cells. This was also associated with upregulation of the expression of several genes coding for DNA and chromatin modification enzymes, such as: *DDM1*, a member of the broad SWI2/SNF2 protein family DNA promoting chromatin remodelling (FC 3.8); cytosine methyltransferase *MET1 *(FC 3.7); histone deacetylases 2A (FC 3.9), 2B(FC 3.3), 2C(FC 2.7), and *HDT4 *(FC 9.5); the histone-lysine N-methyltransferase *SUVH6 *(FC 2.6) and *SUVR2 *(FC 3.7); high-mobility-group *HMG1/2 *family protein (FC 3.8) [78].

Differential expression of epigenetic-related genes suggest that DNA and chromatin modifications occur during the process of habituation to TA or IXB and are possibly involved in maintaining some of the features of TA(-)hab cells. Moreover, the fact that these changes occurred in IXBhab that were still grown in the presence of IXB suggests that DNA and chromatin modifications were initiated when cells were still in the presence of the inhibitor of cellulose synthesis. Whether these changes are related to durable resistance to inhibitors of cellulose synthesis or simply associated with the habituation process is still unknown.

### Other genes of interest

Several other processes were affected in TA(-)hab cells, as many differentially expressed genes were found to be involved in processes like hormone metabolism, transport, stress responses, regulation of transcription, protein modifications and signal transduction (Additional file 2 Table S2 and Additional file 5 Table S5). Most genes involved in biotic stress-related responses were downregulated, and this included genes known to be generally upregulated in response to pathogens, such as pathogenesis-related proteins, several members of disease-resistance family proteins and chitinases. However, expression of several small heat shock factors was upregulated in TA(-)hab cells. Moreover, numerous genes encoding members of different transcription factors families, including WRKY, C2H2-type zinc finger protein, MYB and NAC domain containing proteins, were differentially regulated in TA(-)hab cells, with about half being upregulated and half downregulated. A similar pattern of expression was also found in IXBhab cells, although there were more genes involved in heat shock responses that were downregulated. The fact that many transcription regulator genes are differentially expressed in TA(-)hab cells is not surprising since there are so many different processes that were affected in those cells. These data clearly indicate that the process of habituation is associated with very complex changes in gene expression that certainly alter the general metabolism of the habituated cells.

## Conclusions

Analysis of expression data in poplar TA(-)hab cells demonstrated that durable resistance to inhibitors of cellulose synthesis was linked with a complex reprogramming of gene expression that was associated with expression of epigenetic-related genes. How these changes correlated with resistance to inhibitors of cellulose synthesis remains to be determined. Reprogramming of gene expression could occur in response to inhibition of cellulose synthesis during the habituation process or may be associated with or due to the effect of mutations that enhanced resistance to inhibition of cellulose synthesis. It is also possible that DNA and chromatin modifications were involved in establishing and/or maintaining the resistance to TA. In that case, these changes could theoretically be reverted, resulting in the restoration of cell sensitivity to the inhibitor of cellulose synthesis. However, while the composition of the cell walls of DCB-habituated bean cells cultured in the absence of DCB for several months was almost fully restored to control levels, resistance to high concentrations of DCB was still maintained in DCB-dehabituated cells, suggesting that resistance to DCB cannot be reversed in these cells [43]. Consequently, it is possible that stable and irreversible changes in DNA sequences (mutations) were required for resistance to DCB. Further characterization of the process of habituation to TA will be necessary to determine what changes are essential for the maintenance of resistance to TA. These results will not only be useful to understand how plant cells respond to the toxin, but may provide key information on a wide range of processes, including cellulose synthesis, cell wall organization, intracellular communication between the cell wall and the nucleus, and the activation of epigenetic-related changes in response to inhibition of cellulose synthesis.

## Methods

### Plant material and treatments

Hybrid poplar cell suspensions (*Populus trichocarpa *x *Populus deltoides *H11-11) maintained in the dark in Murashige and Skoog (MS) medium (pH 5.7) supplemented with B5 vitamins [80] were subcultured weekly in a 1:4 dilution or biweekly for TA-habituated cell suspensions. TA was produced and purified from *Streptomyces scabies *using oat bran broth cultures as described before [5,8]. To study morphological changes and cell death, TA was diluted (10 mM) in methanol and added to cell suspensions at the indicated final concentrations 3 d after subculture, or at the time of transfer for the habituation process. IXB (Crescent Chemicals Co., Inc., Islandia, NY, USA) and DCB (Sigma-Aldrich) both diluted (10 mM) in methanol were added to cell suspensions 3 d after subculture at the indicated final concentrations. Control cells were treated with the same volume of methanol. The final concentration of methanol added to cell suspensions during habituation was always less than 0.014% and had no effect on cell survival or growth.

### Detection of cell death

The number of dead cells was determined by staining the cell cultures 1:1 with 0.4% trypan blue diluted in 140 mM NaCl and 3.5 mM K_2_HPO_4 _as described before [8].

### TA-habituation of poplar cell suspensions

Habituation of poplar cell suspensions to 1.3 μM TA was performed by adding increasing levels of TA at each subculture over a period of 12 months, beginning with 0.1 μM thaxtomin A. From 2 to 4 subcultures were performed between each step-up in TA concentration. Non-habituated hybrid poplar cells used as control received the same volume of methanol as that added to TA-habituated cells. From a concentration of 0.4 μM TA, TA-habituated cells were subcultured every other week due to the reduced growth rate. After 12 months, half of the cells adapted to 1.3 μM of thaxtomin A were transferred to culture medium without TA. These cells are referred to as "TA(-)hab" cells. These cell suspensions were subcultured every two weeks in the absence of TA for an additional period of at least 18 months (more than 40 subcultures) before performing analyses.

### Cell wall purification

Suspension-cultured poplar cells were harvested by filtration 24 h after adding TA or methanol (control) and 10 d after subculture for TA(-)hab cells and non-habituated cells. Cells were quickly frozen in liquid nitrogen and kept at -80°C until further used. Plant cell walls were extracted as described [31]. In brief, cells were washed with potassium phosphate buffer, homogenized and treated with 2.5 units ml^-1 ^of α-amylase (Sigma-Aldrich) for 4 h at 37°C. After centrifugation, the pellets were sequentially washed with potassium phosphate buffer, distilled water, acetone, methanol:chloroform and dietylether and air-dried.

### Sugar quantification

Sugars were quantified from cell wall extracts or from crystalline cellulose fraction purified with a modified protocol from Updegraff (1969) [81]. Briefly, dry cell wall samples were boiled for 1 h in acetic-nitric reagents. The acid insoluble fraction (crystalline cellulose) was recovered on a glass filter (GF/C, 2.5 cm diameter, Whatman) and washed thrice with distilled water. Hydrolysis of whole cell wall extracts or crystalline cellulose fraction was performed as described by Ruiz and Ehrman (1996) [82]. In summary, 3 mL sulfuric acid (72%) was added to dry cell wall extracts or to the acid-insoluble fraction on the glass filter. Samples were incubated for 2 h in a 30°C water bath, diluted to a final concentration of 4% sulfuric acid and autoclaved at 121°C for 1 h. Fucose was added as an internal control and samples were filtered through a 0.45 μM nylon filter. Monosaccharides were quantified using a Dionex DX 500 HPLC-system equipped with an ED40 electrochemical detector and a CarboPac PA10 ion exchange column. Extraction of uronic acid was realized following the technique described by Redgwell and Selvendran (1986) [83]. Briefly, dry cell walls were incubated at room temperature with 50 mM cyclohexane-trans-1,2-diaminetetra-acetic acid sodium salt (CDTA) at pH 6.5 for 6 h. After centrifugation, the supernatant (CDTA-1 fraction) was preserved and the residue was extracted with CDTA for 2 h (CDTA-2 fraction). The CDTA-1 and CDTA-2 fractions were combined, filtered through a glass fibre filter, dialyzed and lyophilized. Uronic acid content of cell wall from the CDTA fractions was determined by the m-hydroxydiphenyl colorimetric assay of Filisetti-Cozzi and Carpita (1991) [84]. The uronic acid lyophilized samples were suspended in 0.4 mL water, to which are added 40 μL of 4 M sulfamic acid/potassium sulfamate solution and 2.4 mL sulfuric acid. This mixture was heated at 100°C for 20 min and cooled on ice. Thereafter, 80 μL of 0.15% m-hydroxydiphenyl diluted in 0.5% sodium hydroxide was added and mixed vigorously. After a 10-min incubation, absorbance was measured at 525 nm and the uronic acid content was estimated by comparison with a standard calibration curve of galacturonic acid.

### Confocal and electronic microscopy

Cells examined by confocal microscopy were stained 1:1 with 0.01% fluorescein diacetate (Sigma), a fluorescent marker for cell viability. Confocal laser-scanning microscopy was performed with an Olympus microscope 1X70 equipped with an argon Fluoview laser. Cells observed by electron microscopy were fixed with 2% glutaraldehyde and 4% paraformaldehyde (buffered with 10 mM cacodylate, pH 7.4) for 4 h and post-fixed with 1% osmium tetroxide for 3 h. The specimens were dehydrated through ethanol series (30-50-70-80-90-100%) and embedded with Epon. Samples were examined with a Philips model 201 electron microscope.

### Sample preparation for microarray analysis, data collection and analyses

Each sample was taken from an individual flask of non-habituated poplar cells or TA(-)hab cells grown for 5 d after subculture. Total RNA was extracted as previously described [8]. RNA quality assessment, synthesis of cRNA, labeling and hybridizations to Affymetix GeneChip Poplar Genome Array were performed at Genome Québec, Innovation Center (McGill University, Montréal, Canada) following Affymetrix recommended protocols. Six arrays were hybridized, representing 3 arrays per cell type. Data and statistical analyses were carried out using FlexArray 1.3 [46]. Raw signal intensities were normalized using Robust Multi-array Average methodology (RMA) [45] and Significance analysis of microarrays (SMA) [85] was performed to determine the differentially expressed genes. Raw data obtained for IXB-habituated cells (GSE6181 or NASCARRAYS-27) were also analyzed using RMA and SAM with the FlexArray software. Probesets corresponding to genes in TA(-)hab cells that had > 2.5 FC in expression with a *P *value < 0.05 when compared to non-habituated levels were selected. All materials and procedures complied with the MIAME standards set for microarray data [86]. The full dataset has been submitted to the Gene expression omnibus (GEO) and is available through GEO Series accession number GSE17804.

Gene annotation was performed using the PLEXdb database [47]http://www.plexdb.org, PopArray database http://aspendb.uga.edu/poparray[48] and NetAffx analysis from the Affymetrix website http://www.affymetrix.com. Ambiguous annotations were further confirmed using BLAST similarity searches. For gene models associated with more than one probeset (Additional file 3 Table S3), only one probeset with the highest FC was selected for other analyses (Additional file 2 Table S2). Predicted gene ontology (GO) for each probeset was obtained using the AgriGO analysis tool http://bioinfo.cau.edu.cn/agriGO/[49]. The MapMan 2.2 software [50] was used to visualize changes in expression in different metabolic pathways. A custom-made mapping file based on differentially expressed genes in TA(-)hab cells was assembled using information from the poplar Ptrich_AFFY_09 mapping file that we updated with the most recent annotations.

### qPCR validation of microarray results

Five upregulated and five downregulated genes in TA(-)hab cells according to the microarray analyses were randomly selected for quantitative PCR (qPCR) validation of microarray results. Total RNA (from the same samples used for microarrays analysis) was treated with Turbo DNA-free (Ambion, Austin, TX) to degrade genomic DNA. Reverse transcription was performed on 2 μg RNA as follows. RNA was mixed with 1 μg oligo(dT)_15 _primer (Promega, Madison, WI) in a volume of 10 μL and incubated at 70°C for 5 min. Reverse transcription was achieved in a total volume of 25 μL after addition of dNTP (final conc. 1 mM), 15 U AMV reverse transcriptase (Promega) and 40 U RNasin (Promega) and incubation at 42°C for 1 h. Real-time PCR were performed with 2 μL (in a final volume of 20 μL) of 1:9 diluted cDNA. PCR conditions were 95°C for 3 min followed by 30 cycles at 95°C for 20 s, 55°C for 45 s and 72°C for 20 s. Primers used for validation are listed in Additional file 4 Table S4. Relative gene expression was calculated according to Pfaffl (2001)[87] using *act11 *as the reference gene [88]. Average FC in TA(-)hab cells of the genes under study were log_2_-transformed and plotted with log_2_-transformed FC in Affymetrix GeneChip [58].

## Authors' contributions

VB carried out the habituation experiment, resistance assays and sugar quantification and help to draft the manuscript; MGM carried out the pectin quantification, resistance assays, transcriptional analysis and help to draft the manuscript; ID participated in the conception of the study and carried out the first habituation experiments and resistance assays; SL carried out the qPCR validation of microarray results; GG carried out the microscopy analyses; OD participated in the first habituation experiments and resistance assays; CB participated in the design of the study and coordination; NB conceived the study, participated in its design, carried out some of the transcriptional analysis, annotation and drafted the manuscript. All authors read and approved the final manuscript.

## Supplementary Material

Additional file 1 **Fig. S1 to S6 and Table S1**. **Fig. S1**. Induction of cell death and DNA fragmentation in hybrid poplar cells and TA(-)hab cells in response to TA. **Fig. S2**. Percentage of monosaccharides in relation to total sugars in different cell types. **Fig. S3**. Microscopic observations of pectic components visualized by ruthenium red staining. **Fig. S4**. MapMan overview of significant changes in expression (> 2.0 FC) for genes associated with metabolism in IXBhab cells. **Fig. S5**. MapMan overview of significant changes in expression (> 2.5 FC) in TA(-)hab cells for genes involved in regulation and cellular responses. **Fig. S6**. MapMan overview of significant changes in expression (> 2.0 FC) in IXBhab cells for genes involved in regulation and cellular responses. **Table S1**. Dimensions of non-habituated hybrid poplar cells (Non-hab), TA-habituated cells (TAhab) cultured with 1.9 μM TA and TA-dehabituated cells (TA(-)hab). Click here for file

Additional file 2 **Table S2 - Fold-change (FC) in gene expression in TA(-)hab hybrid poplar cells compared with non-habituated cells detected using Affymetrix GeneChip Poplar Genome Array**. Click here for file

Additional file 3 **Table S3 - Fold-change (FC) in gene expression for all probesets in TA(-)hab hybrid poplar cells compared with non-habituated cells detected using Affymetrix GeneChip Poplar Genome Array**. Click here for file

Additional file 4 **Table S4 - Validation of GeneChip results by qPCR**. Click here for file

Additional file 5 **Table S5 - Fold-change (FC) in gene expression in IXBhab Arabidopsis cells (Manfield et al., 2004) compared with control cells**. Click here for file
