# Supplementary material for: Habituation to thaxtomin A in hybrid poplar cell suspensions provides enhanced and durable resistance to inhibitors of cellulose synthesis
Source: BMC Plant Biol. 2010 Dec 10;10:272. doi: 10.1186/1471-2229-10-272 (PMC3016406; doi:10.1186/1471-2229-10-272)
Supplement: Additional file 1 — Fig. S1 to S6 and Table S1. Fig. S1. Induction of cell death and DNA fragmentation in hybrid poplar cells and TA(-)hab cells in response to TA. Fig. S2. Percentage of monosaccharides in relation to total sugars in different cell types. Fig. S3. Microscopic observations of pectic components visualized by ruthenium red staining. Fig. S4. MapMan overview of significant changes in expression (> 2.0 FC) for genes associated with metabolism in IXBhab cells. Fig. S5. MapMan overview of significant changes in expression (> 2.5 FC) in TA(-)hab cells for genes involved in regulation and cellular responses. Fig. S6. MapMan overview of significant changes in expression (> 2.0 FC) in IXBhab cells for genes involved in regulation and cellular responses. Table S1. Dimensions of non-habituated hybrid poplar cells (Non-hab), TA-habituated cells (TAhab) cultured with 1.9 μM TA and TA-dehabituated cells (TA(-)hab). [file 1471-2229-10-272-S1.PDF]

Additional file 1

Figure Legends

Fig. S1. Induction of cell death and DNA fragmentation in hybrid poplar cells and TA (-)hab cells in response to TA.

A-B Detection of cell death by trypan blue staining in hybrid poplar cell suspensions treated for 48 h with: **A** methanol (control) **B** TA (2.0 µM).

C-D Detection of cell death by trypan blue staining in TA(-)hab cells treated for 48 h with: **C** methanol (control) **D** TA (2.0 µM).

E-F Detection of DNA fragmentation by TUNEL assay (green) in hybrid poplar cell suspensions treated for 72 h with: **E** methanol (control) **F** TA (2.0 µM). Nuclei were stained with DAPI (blue).

G-H Detection of DNA fragmentation using TUNEL assay in TA(-)hab cells treated for 72 h with: **G** methanol (control) **H** TA (2.0 µM). Nuclei were stained with DAPI (blue).

I Percentage of cell death (detected by trypan blue staining) and TUNEL positive cells induced by TA (2.0 µM) in hybrid poplar cells (control) and TA(-)hab cells after 24 h, 48 h and 72 h. Values represent means (±SD) of two independent experiments including at least 500 cells each. Percentage of cell death and TUNEL positive cells in methanol-treated cells was always less than 10% (data not shown).

Fig. S2. Percentage of monosaccharides in relation to total sugars in different cell types. Non-hab: non-habituated hybrid poplar cells; TA(-)hab: TA-habituated hybrid poplar cells without TA. Values represent the means of three independent experiments ± SD.

Fig. S3. Microscopic observations of pectic components visualized by ruthenium red staining. **A** non-habituated hybrid poplar cells, **B** TA-habituated cells without TA.

Fig. S4. MapMan overview of significant changes in expression (>2.0 FC) for genes associated with metabolism in IXBhab cells.

Fig. S5. MapMan overview of significant changes in expression (>2.5 FC) in TA(-)hab cells for genes involved in **A** regulation **B** cellular responses.

Fig. S6. MapMan overview of significant changes in expression (>2.0 FC) in IXBhab cells for genes involved in **A** regulation **B** cellular responses.

Table S1. Dimensions of non-habituated hybrid poplar cells (Non-hab), TA-habituated cells (TAhab) cultured with 1.9 µM TA and TA-dehabituated cells (TA(-)hab).

Additional Materials and Methods

Measurement of cellular dimensions

Cell measurements [diameters, area (A), perimeter (P)] were taken from 100 cells using the Zeiss application software AxioVision LE V4.5 with a Zeiss AxioImager Z1 microscope. The circularity shape factor (C) was determined using $C = 4\pi A/P^2$. Values of C range between 0 and 1, with values closer to 0 indicating very elongated cells and values closer to 1 for circular objects (Encina et al., 2002).

Cell death and TUNEL assays

Cell death and TUNEL assays were performed as described in Duval et al., 2005.

Coloration of pectins

Cells were mixed 1:1 with ruthenium red (0.02%) for 2 to 5 min before microscopic observation.

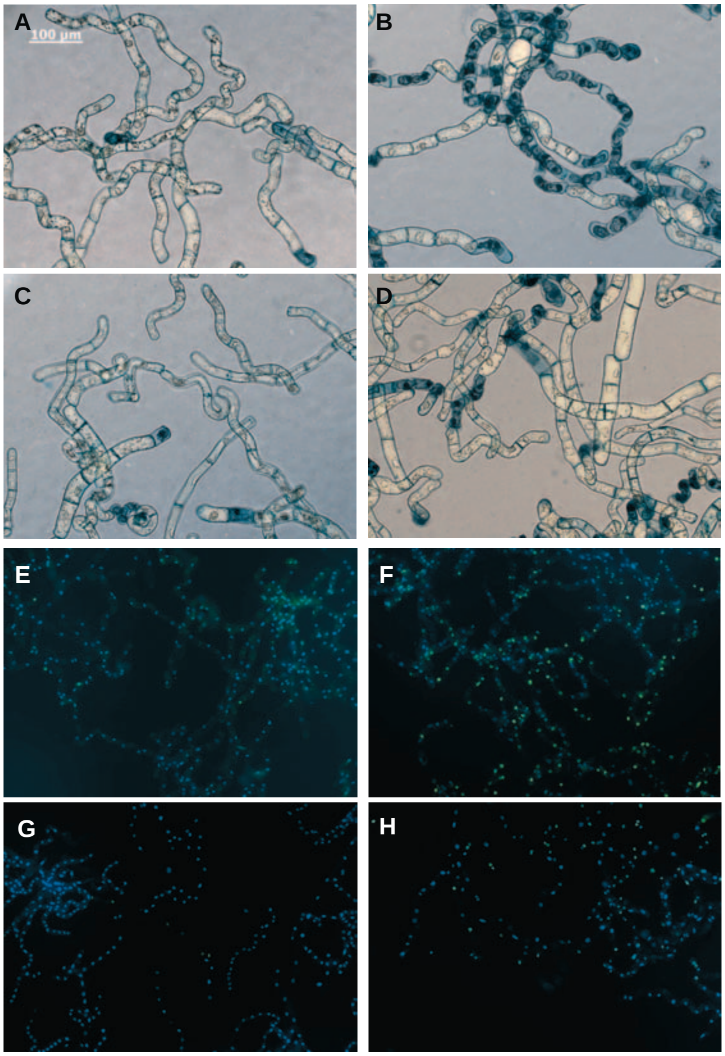

I)

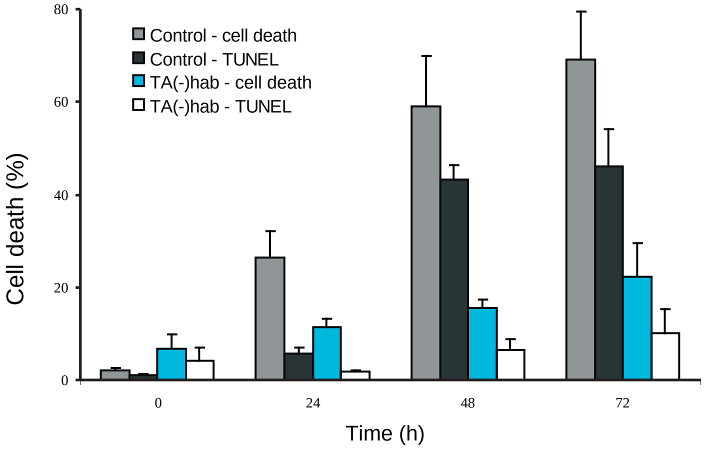

Fig. S1. Induction of cell death and DNA fragmentation in hybrid poplar cells and TA(-)hab cells in response to TA.

A-B Detection of cell death by trypan blue staining in hybrid poplar cell suspensions treated for 48 h with: **A** methanol (control); **B** TA (2.0 µM).
C-D Detection of cell death by trypan blue staining in TA(-)hab cells treated for 48 h with: **C** methanol (control); **D** TA (2.0 µM).
E-F Detection of DNA fragmentation by TUNEL assay (green) in hybrid poplar cell suspensions treated for 72 h with **E** methanol (control) **F** TA (2.0 µM). Nuclei were stained with DAPI (blue).
G-H Detection of DNA fragmentation using TUNEL assay in TA(-)hab cells treated for 72 h with **G** methanol (control); **H** TA (2.0 µM). Nuclei were stained with DAPI (blue).
I Percentage of cell death (detected by trypan blue staining) and TUNEL positive cells induced by TA (2.0 µM) in hybrid poplar cells (control) and TA(-)hab cells after 24 h, 48 h and 72 h. Values represent means (±SD) of two independent experiments including at least 500 cells each. Percentage of cell death and TUNEL positive cells in methanol-treated cells was always less than 10% (data not shown).

Fig. S1, Brochu et al. 2010

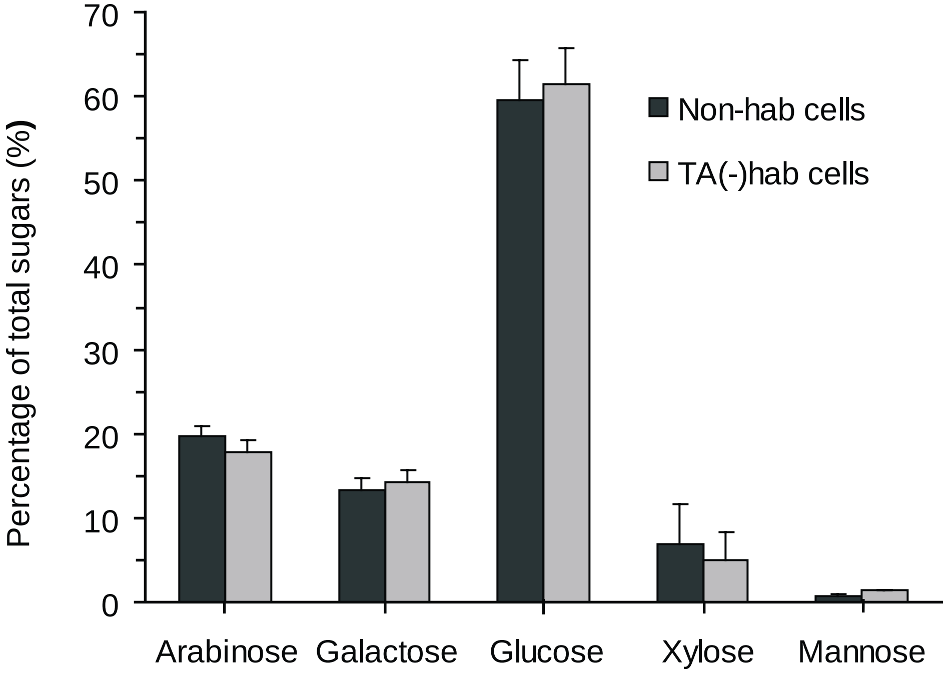

Fig. S2. Percentage of monosaccharides in relation to total sugars in different cell types. Non-hab: non-habituated hybrid poplar cells; TA(-)hab: TA-habituated hybrid poplar cells without TA. Values represent the means of three independant experiments ± SD.

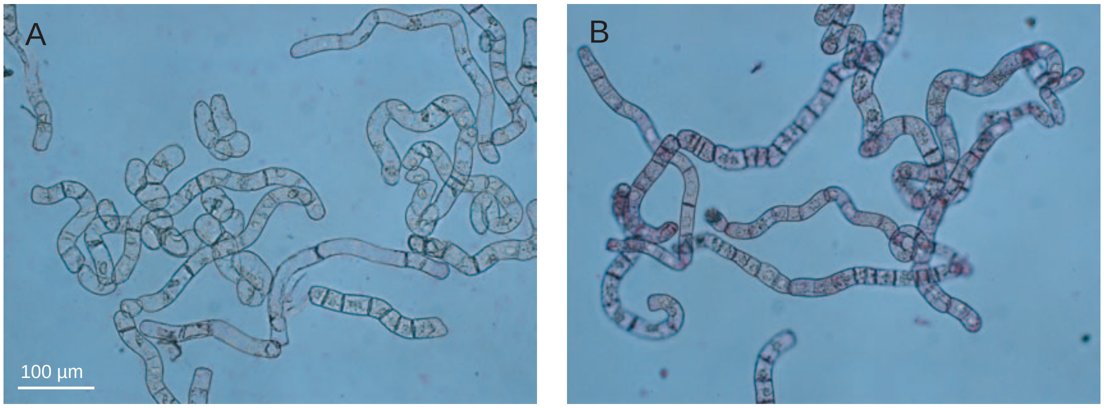

Fig. S3. Microscopic observations of pectic components visualized by ruthenium red staining. **A** Non-habituated hybrid poplar cells, **B** TA-habituated cells without TA (TA(-) hab).

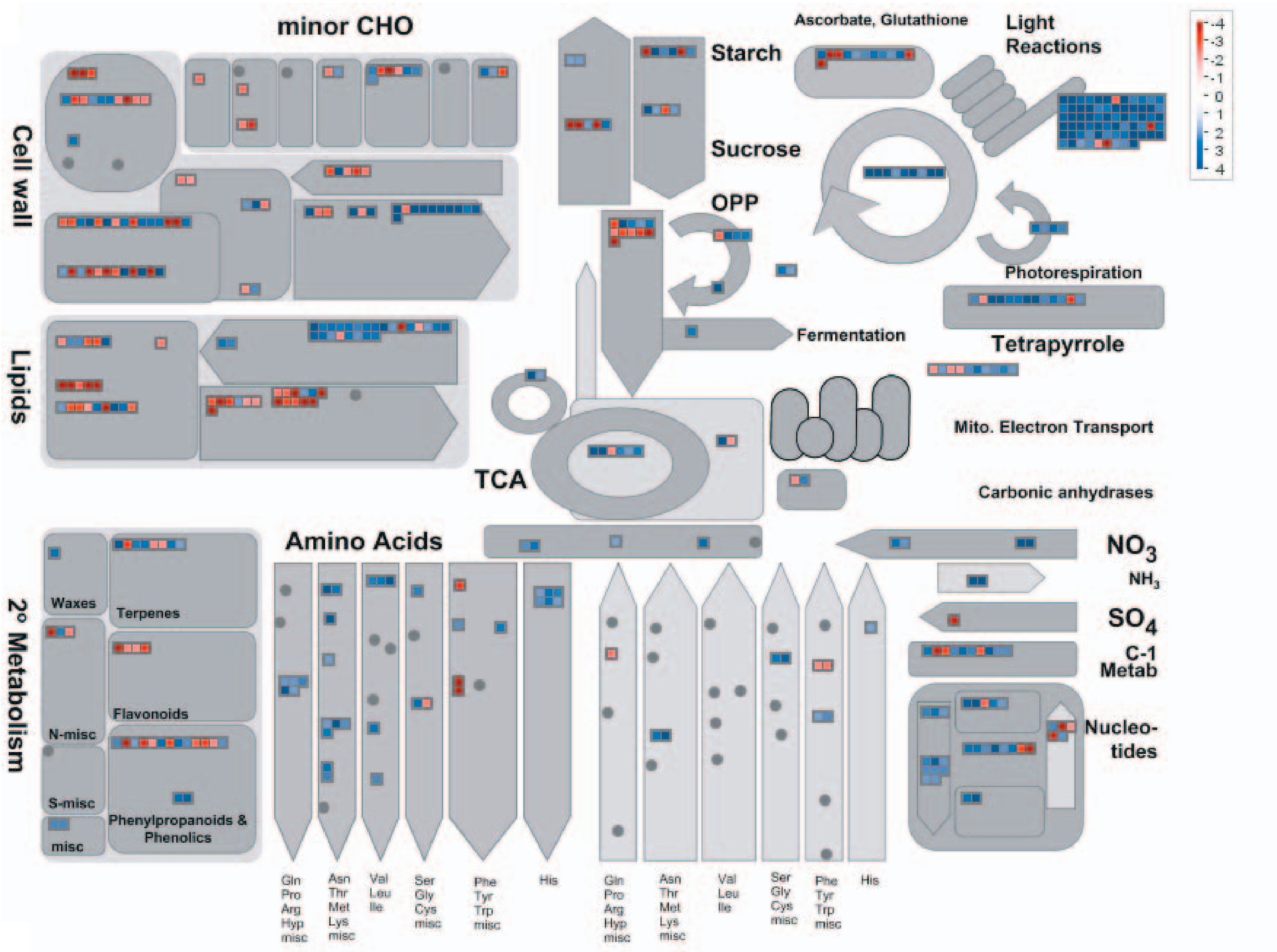

Fig. S4. MapMan overview of significant changes in expression (>2.0 FC) for genes associated with metabolism in IXBhab cells.

Fig. S4, Brochu et al. 2010

A)

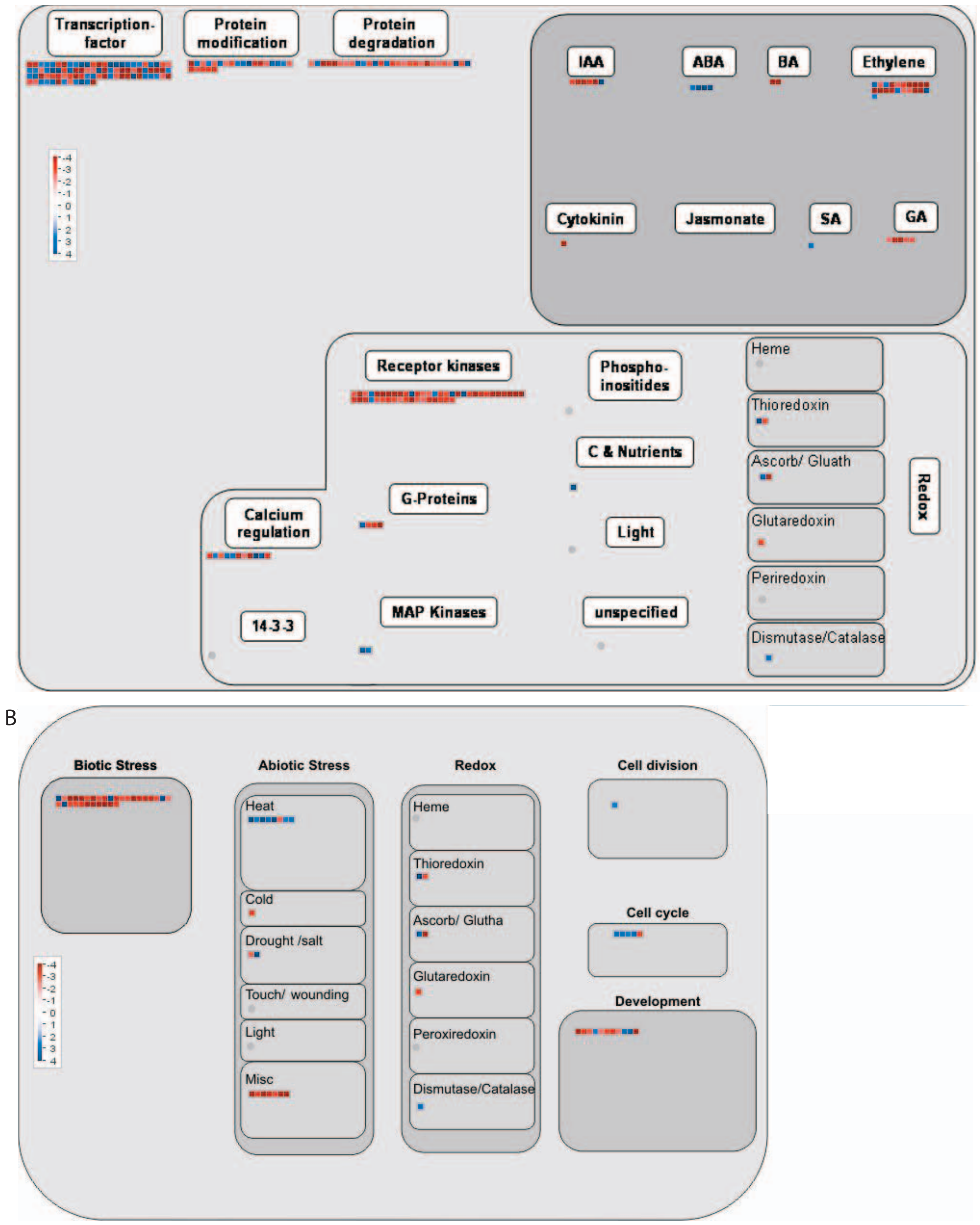

Fig. S5. MapMan overview of significant changes in expression (>2.5 FC) in TA(-)hab cells for genes involved in: **A** regulation **B** cellular responses.

Fig. S5, Brochu et al. 2010

A)

B)

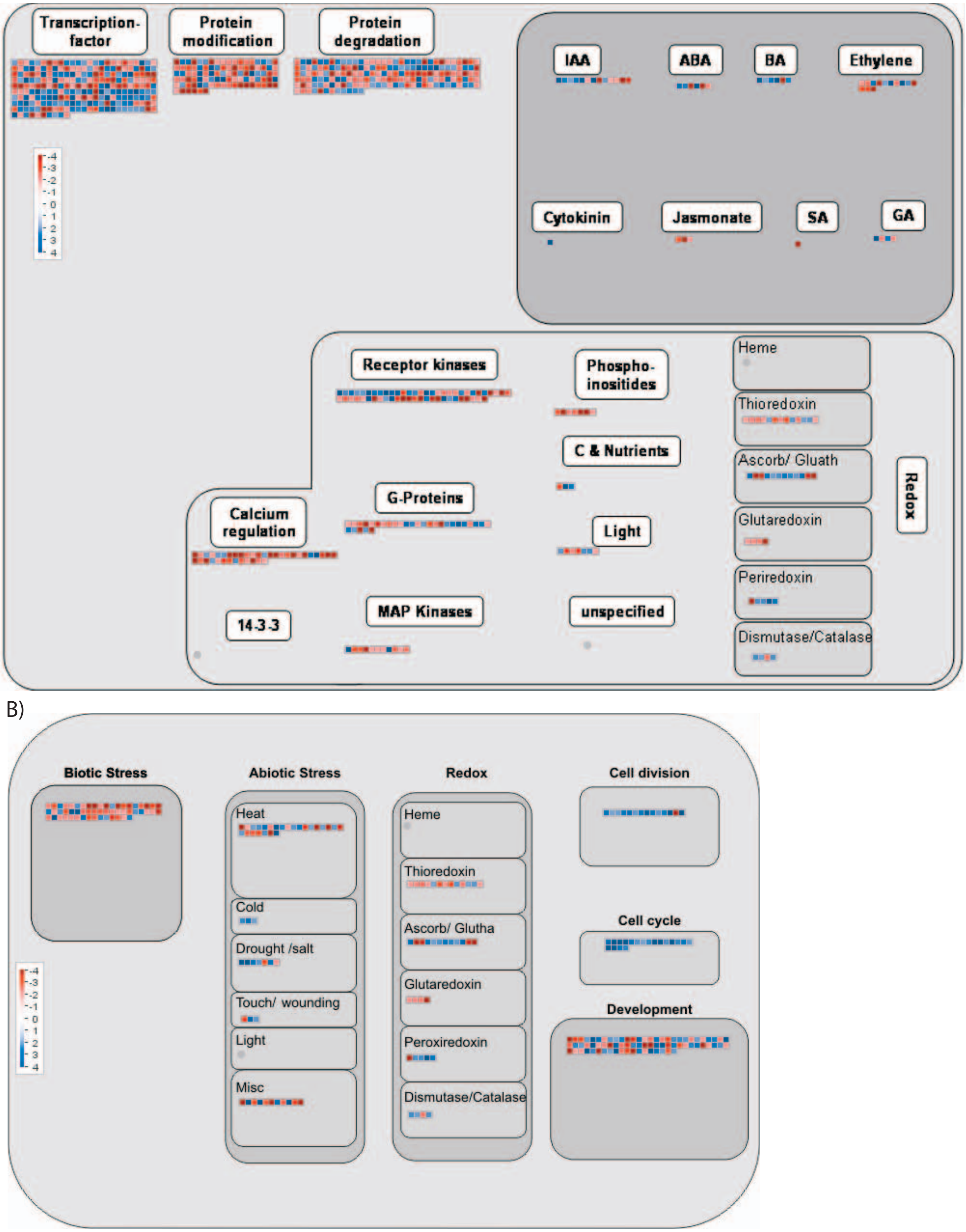

Fig. S6. MapMan overview of significant changes in expression (>2.0 FC) in IXBhab cells for genes involved in: **A** regulation **B** cellular responses.

Table S1. Dimensions of non-habituated hybrid poplar cells (Non-hab), TA-habituated cells (TAhab) cultured with 1.9 µM TA and TA-dehabituated cells (TA(-)hab).

Cell type	Maximum diameter (µM)	Minimum diameter (µM)	Circularity shape factor
Non-hab	$53.2 \pm 19.7^{a,b}$	22.3 ± 3.2^{a}	0.66 ± 0.09^{a}
TAhab	49.2 ± 17.5^{a}	31.1 ± 7.2^{b}	0.84 ± 0.09^{b}
TA(-)hab	64.0 ± 21.6^{b}	17.9 ± 3.9^{c}	0.56 ± 0.12^{c}

Values are the means ± SD of measurements taken from 100 cells. Statistically different values (Student's *t*-test , $P < 0.05$) are indicated with a different letter in a column.
